# Intraoperative radiotherapy might not serve as a standard therapy for retroperitoneal liposarcoma: insights from a population-based propensity score-matched study

**DOI:** 10.3389/fonc.2024.1431920

**Published:** 2024-10-25

**Authors:** Xiao Zhou, Aobo Zhuang, Xi Li, Zhe Xi, Yingxue Cheng, Guangting Yan, Yue Wang, Gen Zhang, Yangyang Huang, Chenhe Zhang, Fuan Xie, Xin Ma, Ting Wu, Wengang Li

**Affiliations:** ^1^ Cancer Research Center, School of Medicine, Xiamen University, Xiamen, China; ^2^ Department of Hepatobiliary Surgery, Xiang’an Hospital of Xiamen University, School of Medicine, Xiamen University, Xiamen, China; ^3^ School of Public Health, Harvard University, Boston, MA, United States

**Keywords:** retroperitoneal liposarcoma, intraoperative radiotherapy, SEER, propensity score matching, overall survival

## Abstract

**Background:**

Difficulty in achieving complete resection leads to a poor prognosis for retroperitoneal soft tissue sarcoma, hence emphasizing the significance of adjuvant treatment. The benefit of preoperative radiotherapy for retroperitoneal liposarcoma was initially demonstrated by the STRASS trial. However, the impact of intraoperative radiotherapy (IORT) on retroperitoneal liposarcoma remains unexplored.

**Method:**

Patients with retroperitoneal liposarcoma were identified in the Surveillance, Epidemiology, and End Results (SEER) database, treated between 2000 and 2019. Subsequently, a 1:1 propensity score-matched (PSM) analysis was conducted based on variables identified from a multivariate analysis. T-tests were used to assess differences in normally distributed continuous variables, while the rank-sum test was applied to variables that did not follow a normal distribution. The chi-squared test was utilized to evaluate differences in categorical variables. Ultimately, survival analysis was performed using SPSS to evaluate patient prognosis.

**Result:**

A total of 2129 patients with retroperitoneal liposarcoma were included in our study. Age, sex, histology, grading, chemotherapy, and tumor size as independent prognostic risk factors for these patients through multivariate Cox regression analysis. Subsequently, 66 patients were included in the survival analysis through PSM, with 33 patients receiving IORT. Finally, the survival analysis revealed that there was no difference in overall survival among patients with retroperitoneal liposarcoma, regardless of whether they received IORT or not (p= 0.711).

**Conclusion:**

As an exploratory study, our findings suggest that patients may not derive benefit from intraoperative radiotherapy. These observations are intended to lay the groundwork for future prospective clinical studies.

## Background

Soft tissue sarcomas (STS) are heterogeneous tumors that arise from mesenchymal cells, including muscle, fat, cartilage, nerve, and vascular tissue. Consequently, STS occur in all body parts, with a higher prevalence in the lower and upper limbs, and a comparatively lower prevalence in the retroperitoneum, chest wall, and head and neck (1). STS accounts for approximately 1% of all newly diagnosed malignant solid tumors, equating to approximately 12,000 cases annually in the United States (2). Despite the low incidence rate of STS, retroperitoneal sarcoma (RPS) still contributes to approximately 15% of all STS cases, with an average annual incidence of 2.7 per million people (3). Among adults, the most common histological type is liposarcoma (approximately 50-70%), which is further subdivided into well-differentiated liposarcoma (WDLPS) (synonymous with atypical lipoma tumors [ALT] when diagnosed in the extremities) and dedifferentiated liposarcoma (DDLPS) (4).

Local area recurrence (LAR) is the dominant form of recurrence in patients with RPS and often leads to death (5). Therefore, reducing LAR is an important goal for patients with RPS (5). The primary and only treatment for localized RPS is surgical excision, with the major oncological goal being to achieve complete resection (R0+R1) (6).

Although many people have undergone multiple sequential excisions of multiple organs, the outcomes of RPS are generally less satisfactory than those of other soft tissue sarcomas (7). Hence, adjuvant treatment with surgery holds significance; nonetheless, there is insufficient evidence to support the effectiveness of chemotherapy in retroperitoneal liposarcoma (RPL) (6). Surgeons have, therefore, begun to experiment with radiotherapy. Multiple randomized trials have confirmed that preoperative or postoperative radiotherapy during limb-sparing surgery significantly reduces the risk of local recurrence (LR) in patients with STS in the extremities (5). In recent years, there has been a growing trend among academics and experts to utilize preoperative radiotherapy as a prominent approach in the treatment of RPS. The STRASS trial was also the first to demonstrate the benefits of preoperative radiotherapy for RPL (8).

An advantage of intraoperative radiotherapy (IORT) is the ability of surgeon to remove critical organs and attempt to irradiate only the tumor bed. This advantage allows the dose to be selectively increased in the risk area, thereby increasing the treatment ratio between target and normal tissues (5). However, research into IORT in patients with retroperitoneal soft tissue sarcoma needs to be improved, and the number of patients included in the prospective only studies must be expanded (5).

Although the STRASS trial demonstrated that patients with RPL may benefit from neoradiotherapy, there are currently no studies on the prognostic impact of IORT in patients with RPL. Therefore, we conducted the exploratory study by reviewing bulk data through searching the Seer database to address the gaps in IORT of RPL patients and provide direction for further prospective clinical research.

## Method

**Figure 1:**
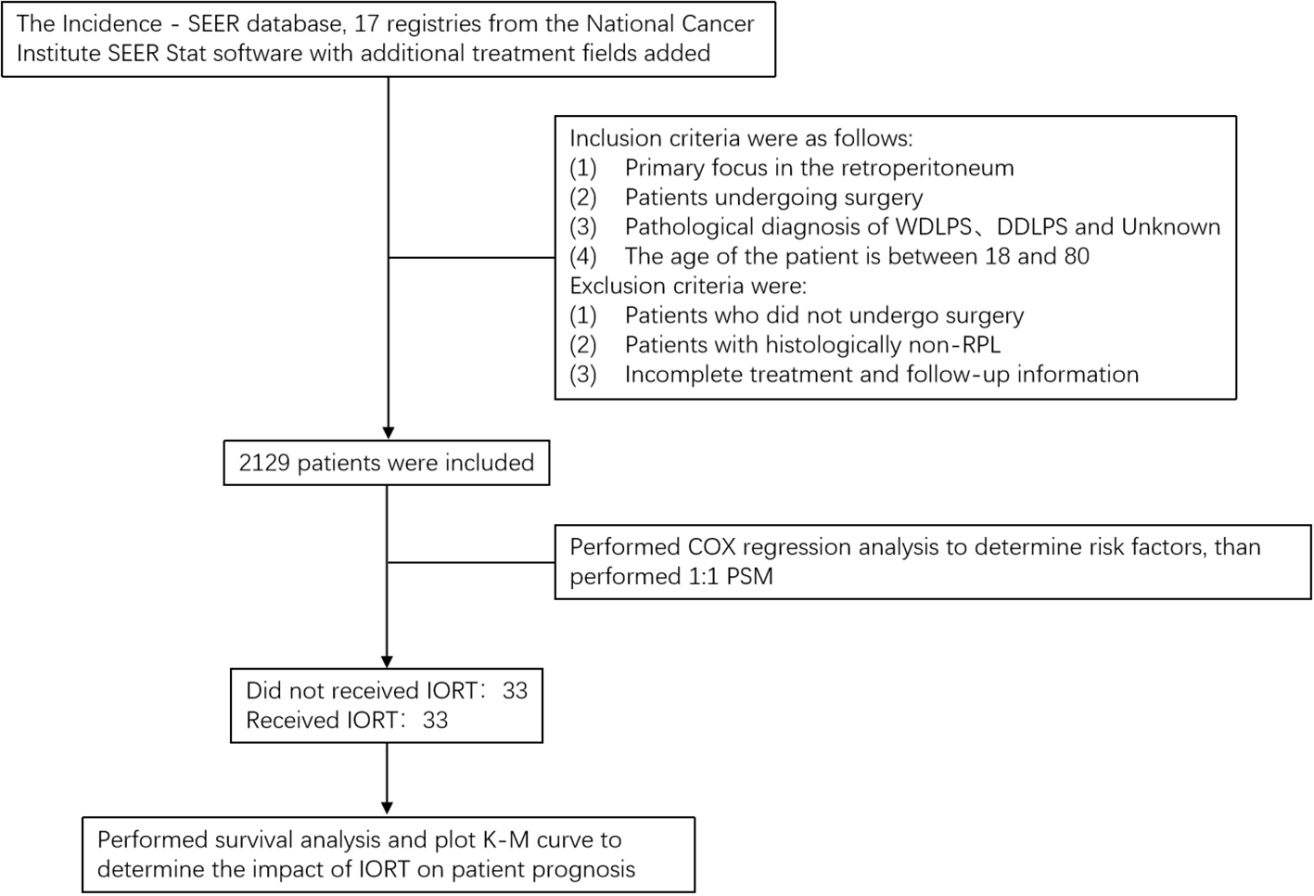
Research flowchart.

According to the **Figure 1**, patient data were collected from the Incidence - SEER database, 17 registries from the National Cancer Institute SEER Stat software with additional treatment fields added. According to the third edition of the International Classification of Diseases for Oncology (ICD-O-3), patients diagnosed with RPL and underwent surgery in 2000 and 2019 were incorporated into the study cohort. Inclusion criteria were as follows: (1) primary focus in the retroperitoneum; (2) patients undergoing surgery; (3) pathological diagnosis with well-differentiated liposarcoma, dedifferentiated liposarcoma, and unknown; (4) The age of the patient is between 18 and 80. Exclusion criteria were: (1) patients who did not undergo surgery; (2) patients with histologically non-RPL; (3) incomplete treatment and follow-up information. The variables chosen for analysis include the year of diagnosis, age, sex, race (Caucasians, African-Americans, or other), site code ICD-0-3, tissue grade, histology record ICD-0-3, radiotherapy (sequence of radiotherapy with surgery), status of lymph node dissection, chemotherapy (whether or not), month of survival, COD to site rec KM, and vital status record.

The characteristics that are extracted include year of diagnosis, age, sex, race, histology, grade, radiotherapy, lymph node dissection status, chemotherapy, and tumor size. Univariate Cox regression analysis is conducted on the entire cohort, and multivariate Cox regression analysis is performed on statistically significant variables to evaluate the impact of variables on the prognosis of RPL patients.

As this study is a retrospective analysis using the SEER database, the implementation of IORT may be subject to selection bias and potential confounding factors. To address this issue, we balanced the baseline characteristics between different treatment groups through 1:1 propensity score matching (PSM), making the cohorts more comparable and the results more reliable. Using the Cox regression model, we identified key prognostic factors affecting the prognosis of patients with retroperitoneal liposarcoma, such as tumor size, grade, histological type, and patient baseline characteristics (e.g., age, gender, race). Based on these variables, we calculated the propensity score for each patient and employed a 1:1 nearest neighbor matching algorithm, setting the maximum allowable propensity score difference (caliper width) at 0.01, and divided the patients into two groups based on the treatment methods. Subsequently, we validated the matching quality by performing comparative analysis on the matched characteristics to ensure that the baseline features of the two groups were similar.

Overall survival (OS) of RPL is defined as the duration between the initial diagnosis and the occurrence of death from any cause or the most recent follow-up. Using survival status as the dependent variable and treatment modality as the independent variable, 1:1 PSM was performed with a clamp value of 0.01 to calibrate for the effect of baseline clinicopathological differences. The normality test was used to test whether the continuous variables fit the normal distribution (e.g., age, tumor size), the t-test was used to assess whether there were differences between groups for the continuous variables that fit the normal distribution while the rank sum test is used for variables that do not conform to a normal distribution, and the chi-squared test was used to assess whether there were differences between groups for categorical variables (e.g., gender, race, sex, histology, grade, chemotherapy, treatment). Survival analysis was performed by plotting the Kaplan-Meier curve to compare whether there were differences in OS between groups. To eliminate the confounding effects of adjuvant radiotherapy, patients were stratified into two groups based on their exposure to adjuvant radiotherapy (which included preoperative, postoperative, and combined IORT with pre- and postoperative treatments). Subsequently, within these two groups, patients were further matched on a 1:1 PSM based on whether they received IORT as the independent variable. Following this matching, survival analyses were conducted once more to evaluate the outcomes. SPSS (Version 27.0. Armonk, NY: IBM Corp) statistical software was used for statistical analysis and presentation. Double-tailed p < 0.05 was considered statistically significant.

## Result

As shown in **Table 1**, 2129 patients who met the inclusion criteria were included, the age distribution exhibited a median value of 63 (IQR: 54, 71) years and the tumor size distribution had a median value of 208 (IQR: 130, 300) mm. The patient population exhibited minimal disparity in terms of sex, with 1199 male patients and 930 female patients. The most common histological type was dedifferentiation (960 cases, 45.09%), followed by well-differentiation (787 cases, 36.97%), and the most common tumor grade was FNCLCC I (976 cases, 45.84%). Most patients did not receive chemotherapy (1914 cases, 89.9%) and most were Caucasians (1786 cases, 83.89%). There were 18 cases (0.85%) of IORT, 15 cases (0.71%) of Intraoperative radiation with other radiation before/after surgery, 9 cases (0.42%) of preoperative and postoperative radiotherapy, 138 cases (6.48%) of preoperative radiotherapy, 281 cases (13.20%) of postoperative radiotherapy and 1666 cases (78.25%) of no radiotherapy.

**Table 1:** Characteristics of included patients.

Characteristic	Total: 2129
Age	63 (IQR: 54, 71) years
Tumor Size	208 (IQR: 130, 300) mm
Sex:	
Male	1199 (56.32%)
Female	930 (43.68%)
Histology:	
Dedifferentiated	960 (45.09%)
Well-differentiated	787 (36.97%)
Unknown	382 (17.94%)
Grade:	
FNCLCC I	976 (45.84%)
FNCLCC II	396 (18.60%)
FNCLCC III	757 (35.56%)
Chemotherapy:	
Yes	215 (10.1%)
No/unknow	1914 (89.9%)
Race:	
Caucasians	1786 (83.89%)
Other (American Indian/AK Native, Asian/Pacific Islander)	219 (10.27%)
African-Americans	113 (5.31%)
Unknown	11 (0.53%)
Treatment:	
Intraoperative radiation	18 (0.85%)
Intraoperative radiation with other radiation before/after surgery	15 (0.71%)
Radiation before and after surgery	9 (0.42%)
Radiation prior to surgery	138 (6.48%)
Radiation after surgery	281 (13.20%)
Non radiotherapy	1666 (78.25%)
Sequence unknow	2 (0.09%)

As shown in **Table 2**, univariate Cox regression analysis was conducted using variables including year of diagnosis, age, sex, race, histology, grade, radiotherapy, lymph node dissection status, chemotherapy, and tumor size. The study revealed that patients’ prognosis were affected by age (p<0.001), sex (p<0.001), histology (p<0.001), grade (p<0.001), chemotherapy (p<0.001), and tumor size (p= 0.003). Upon inclusion of all aforementioned variables in the multivariate Cox regression analysis, the findings revealed that age (HR = 1.041, 95% CI 1.035-1.047, p<0.001), sex (HR = 1.230, 95% CI 1.080-1.402, p<0. 001), histology (p = 0. 029), grade (p<0.001), chemotherapy (HR = 1.947, 95% CI 1.608-2.357, p<0.001), and tumor size (HR = 1.001, 95% CI 1.000-1.001, p<0.001) were identified as independent risk factors for the prognosis of patients.

**Table 2:** Univariate and Multivariate COX regression analysis.

Characteristics	Univariate analysis		Multivariate analysis	
	P-value	HR (95%CI)	P-value	HR (95%CI)
Age	<0.001	1.040 (1.035-1.046)	<0.001	1.041 (1.035-1.047)
Sex (male vs. female)	<0.001	1.521 (1.337-1.731)	0.002	1.230 (1.080-1.402)
Histology:				
Well-differentiated liposarcoma	<0.001	Reference	0.029	Reference
Dedifferentiated liposarcoma	<0.001	2.615 (2.257-3.029)	0.009	1.362 (1.080-1.718)
Unknown	<0.001	1.477 (1.225-1.780)	0.053	1.227 (0.997-1.510)
Grade:				
FNCLCC I	<0.001	Reference	<0.001	Reference
FNCLCC II	<0.001	1.790 (1.486-2.155)	0.003	1.406 (1.123-1.759)
FNCLCC III	<0.001	2.904 (2.524-3.341)	<0.001	1.979 (1.584-2.472)
TumorSize	0.003	1.000 (1.000-1.001)	<0.001	1.001 (1.000-1.001)
Received chemotherapy	<0.001	2.150 (1.787-2.588)	<0.001	1.947 (1.608-2.357)
Race:				
African-Americans	0.750	Reference		
Other	0.921	1.017 (0.724-1.430)		
Unknown	0.317	0.364 (0.050-2.638)		
Caucasians	0.779	1.042 (0.783-1.386)		
Type of radiotherapy:				
IORT with radiotherapy before/after surgery	0.662	Reference		
IORT	0.460	1.490 (0.517-4.294)		
No radiotherapy	0.295	1.536 (0.688-3.431)		
Radiotherapy after surgery	0.230	1.649 (0.729-3.729)		
Radiotherapy before and after surgery	0.436	0.529 (0.107-2.623)		
Radiotherapy prior to surgery	0.301	1.565 (0.670-3.658)		
Sequence unknown	0.657	1.616 (0.194-13.427)		
Year of diagnosis	0.272	1.007 (0.995-1.019)		
Lymph node dissections:				
0	0.749	Reference		
1	0.905	1.022 (0.713-1.465)		
1~3	0.978	0.997 (0.812-1.225)		
>4	0.170	1.168 (0.936-1.459)		
Unknown	0.975	0.994 (0.699-1.415)		

Due to the significant disparity in the number of patients undergoing IORT relative to the entire cohort, we implemented a 1:1 PSM strategy to eliminate potential confounders by using independent risk factors derived from the previous step of the analysis. After matching, there were no statistically significant differences between the two groups.

Baseline characteristics of the population after PSM are shown in **Table 3**. The median age of patients who underwent IORT was 61 years old, while the median tumor size was 200 mm. The patient population consisted predominantly of individuals exhibiting both well-differentiated and dedifferentiated characteristics, with comparable frequencies observed for each group. The majority of patients exhibited a tumor grade of FNCLCC I. A limited number of patients received chemotherapy. The majority of patients were Caucasians.

**Table 3:** Result of 1:1 propensity score matching.

Characteristics	Did not received IORT (33)	Received any IORT (33)	P-value
Age (years)	62 (IQR: 53-70)	61 (IQR: 53-70)	0.894
Tumor Size (mm)	180 (IQR: 130-260)	200 (IQR: 150-260)	0.572
Sex	Male:16	Male:13	0.323
Histology:			0.439
Well-differentiated	11	15	
Dedifferentiated	16	15	
NOS	6	3	
Grade:			0.393
FNCLCC I	13	17	
FNCLCC II	8	4	
FNCLCC III	12	12	
Chemotherapy:			0.689
Yes	3	4	
No/unknow	30	29	
Race:			0.458
Caucasians	26	29	
Unknown	1	0	
Other	6	4	
African-Americans	0	0	

**Figure 2:**
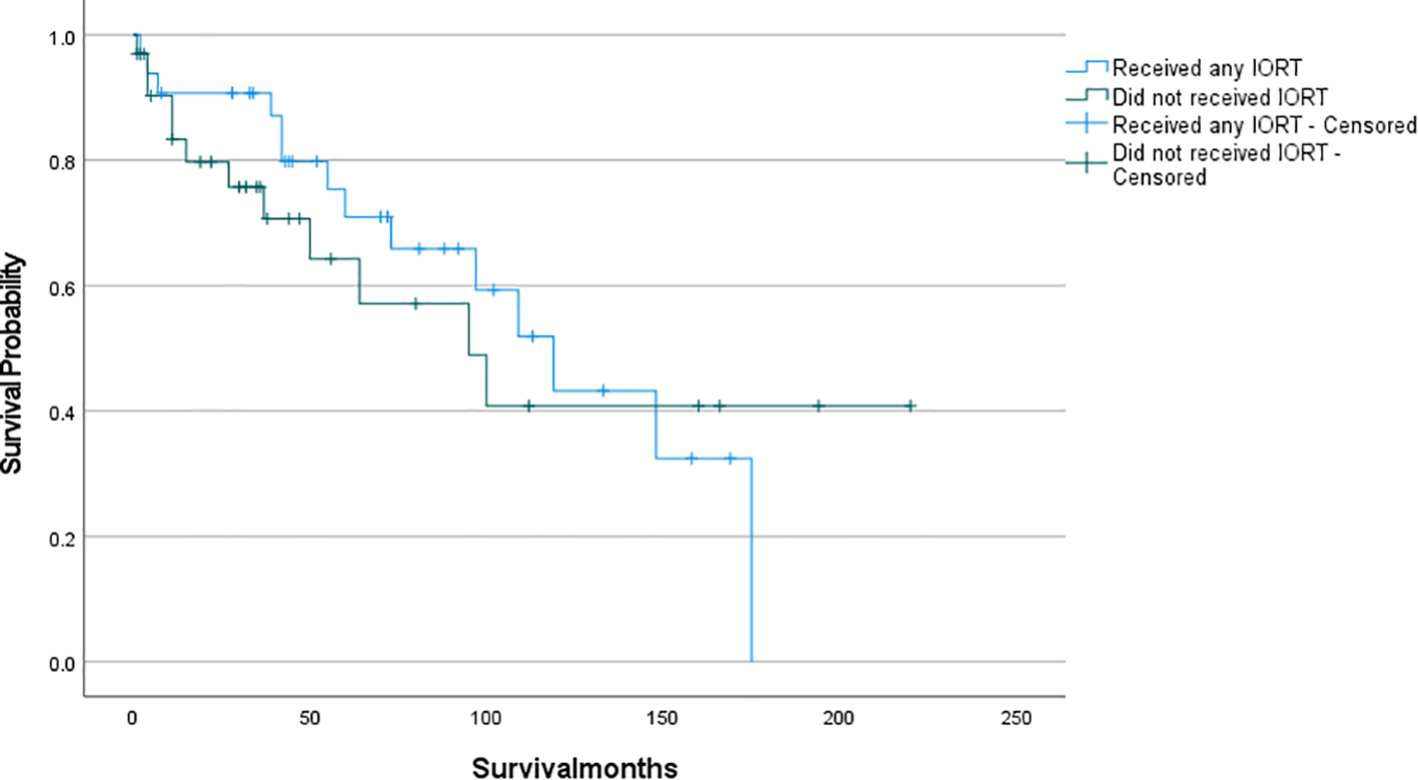
Patients who received IORT compared to those who did not receive IORT (P= 0.711).

**Figure 3:**
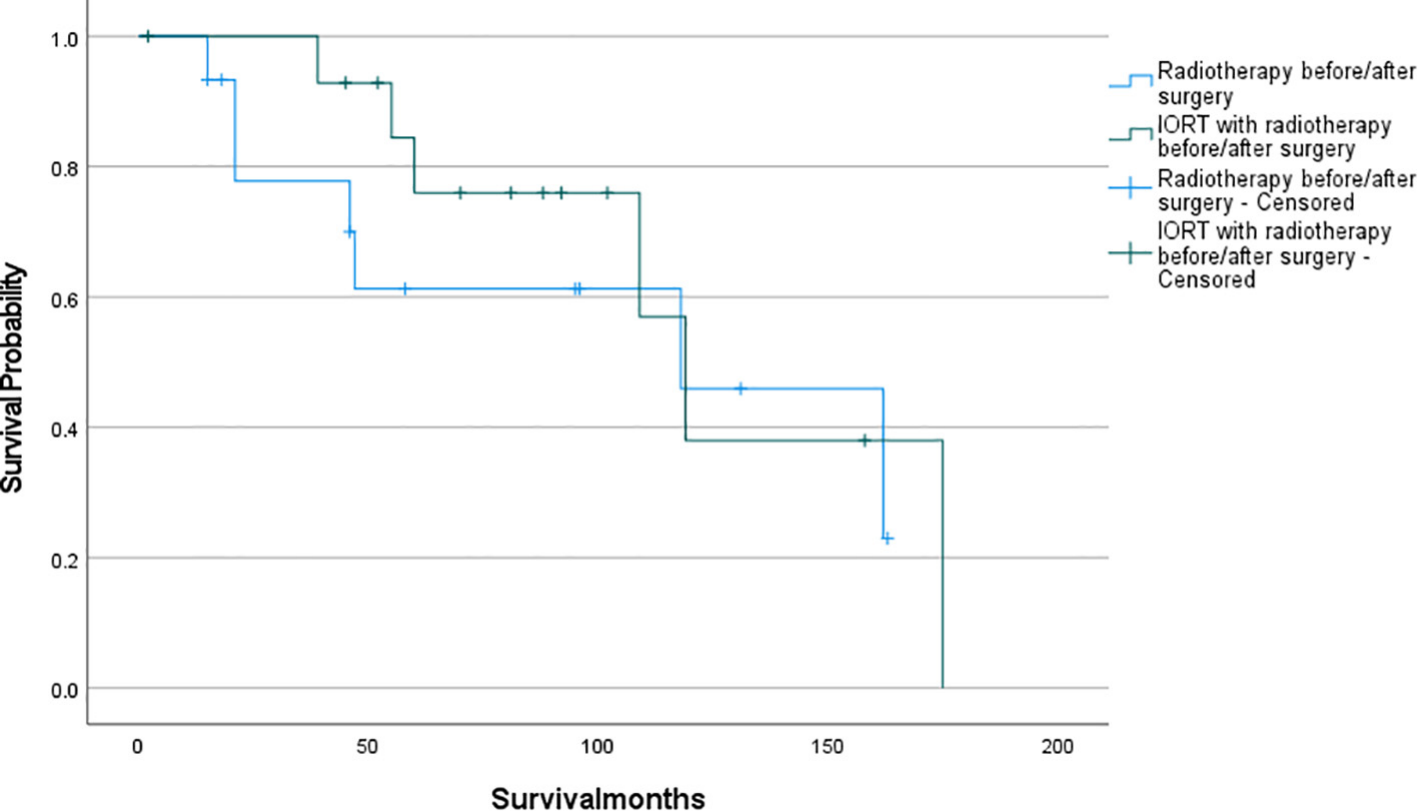
Patients who received IORT with radiotherapy before/after surgery compared to those who received radiotherapy before/after surgery (P= 0.45).

**Figure 4:**
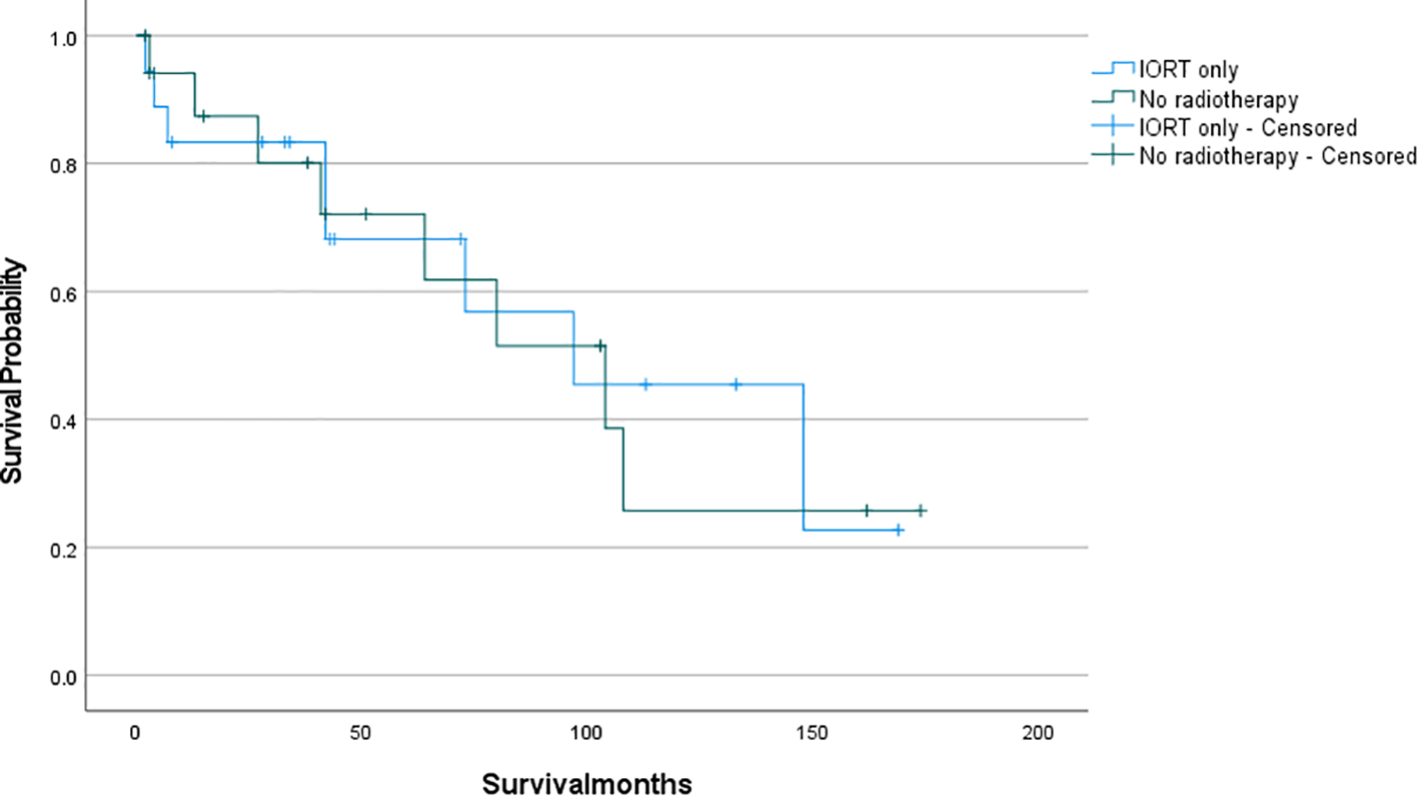
Patients who only received IORT compared to those who did not received radiotherapy (P= 0.899).

The survival analysis was conducted using SPSS software and a survival curve was plotted. According to the Kaplan-Meier analysis, there was no statistically significant disparity in OS between patients who received IORT and those who did not (**Figure 2**: P= 0.711). To eliminate the confounding effects of adjuvant radiotherapy, patients were stratified into two groups based on their exposure to adjuvant radiotherapy (which included preoperative, postoperative, and combined IORT with pre- and postoperative treatments). Subsequently, patients were further matched on a 1:1 PSM based on whether they only received IORT as the independent variable. Following this matching, survival analyses were conducted once more to evaluate the outcomes. And as shown in **Figures 3**, **4**, IORT did not affect patient prognosis, regardless of whether the patient receives adjuvant radiotherapy (P= 0.45, P= 0.899).

## Discussion

Retroperitoneal sarcomas (RPS) frequently lead to local recurrences, which are the primary cause of mortality in affected patients, complete resection is the only means of radical cure, but the effect is not satisfactory (3, 9, 10). There is also a lack of substantial evidence supporting the efficacy of adjunctive chemotherapy (6). And the prognosis of radiotherapy for these patients is also controversial (11–15). In response to these challenges, our study aimed to evaluate the effectiveness of IORT in the treatment of RPL. Our findings revealed that IORT did not significantly improve treatment outcomes.

The effectiveness of preoperative radiotherapy was first demonstrated in the RPL subgroup in the recently published randomized STRASS trial (EORTC 62092/STRASS). This trial reported a notable improvement in 3-year abdominal relapse-free survival in patients who received preoperative radiotherapy (71.6% vs. 60.4%). However, patients diagnosed with leiomyosarcoma (LMS) and high-grade sarcoma were not found to have an increased LR rate when administered preoperative radiotherapy. The findings suggest that preoperative radiotherapy provides a favorable prognosis for patients with this RPL subtype (8).

Callegaro et al. then compared the abdominal relapse-free survival (ARFS) of patients with primary RPS. The study compared the outcomes of patients who were treated with RT in EORTC-STBSG-62092 (STRASS) Phase 3 randomized controlled trial (STRASS cohort) with those who were treated with RT off-trial (STREXIT cohort). The results indicated that the use of radiotherapy improved ARFS in patients with liposarcoma, especially well-differentiated liposarcoma and G1-G2 dedifferentiated liposarcoma. However, radiotherapy did not benefit patients with leiomyosarcoma or G3 dedifferentiated liposarcoma (16).

IORT is a procedure that allows for the administration of high doses of radiation during surgery while the surgeon is removing vital organs and exposing the tumor site (5). The application of IORT has demonstrated prognostic benefits for patients in cases of breast cancer (17). It is a reasonable option to increase the dose and improve local control (LC). The risk of wound healing disorder or gastrointestinal toxicity is minimal. Delivering sufficient radiation over a significant retroperitoneal area without harming other organs poses a considerable challenge regarding RPL. Radiation treatment can have adverse effects on overall survival. Nonetheless, the impact of IORT on the prognosis of patients with RPL remains unknown.

The initial prospective randomized trial aimed to evaluate IORT for retroperitoneal soft tissue sarcoma. A total of 35 patients were enrolled in the trial, with 15 patients received IORT at 20Gy in combination with postoperative radiotherapy at 35-40Gy, and with 20 patients received postoperative radiotherapy at 50-55Gy alone. The results indicated a decrease in local relapses and radiation-related abdominal complications among patients who underwent IORT together with postoperative radiotherapy (18). Their findings is similar to ours, however, there was no significant improvement in overall survival (OS) or cancer-specific survival (CSS) among the patients. Although their study only compared the efficacy of intraoperative radiotherapy (IORT) combined with postoperative radiotherapy versus postoperative radiotherapy alone, and failed to distinguish between the various histologic types. Nonetheless, their research presents novel strategies for implementing IORT in RPS patients.

There has been ongoing debate regarding the efficacy of IORT for sarcoma treatment in previous studies. Wang L. B. et al. found that IORT benefits the OS of liposarcoma patients, which is contrary to our results. This discrepancy may stem from differences in baseline characteristics such as tumor size, race, and grade among patients receiving different treatments in their cohort, as well as a smaller sample size. Our study increased comparability by balancing baseline characteristics between different groups through 1:1 PSM and included a larger sample size, which may explain the differing results (19). Similarly, Gieschen H. L. et al. reported that IORT benefits OS in patients with retroperitoneal sarcoma, but their inclusion of various histological types and a smaller sample of patients receiving IORT (only 16 cases) may have led to different outcomes. Although they found that IORT benefits disease-free survival (DFS) and local control (LC), the differences did not reach statistical significance, possibly due to the small sample size (20). J.-P.E.N. Pierie et al. compared preoperative radiotherapy with combined preoperative and intraoperative radiotherapy and found that the combined treatment improved disease-specific survival and recurrence. Their cohort included 103 patients but did not analyze the effects of using IORT alone, so the benefits of IORT alone in their cohort were still unknown (21). Timothy M. Pawlik et al. analyzed the results of preoperative radiotherapy combined with either IORT or other radiotherapy, finding a 5-year LC rate of 60% and a 5-year OS of 61%, higher than many studies, but similarly did not clearly evaluate the pros and cons of using IORT alone (22). Robert Krempien et al. also found benefits of IORT for retroperitoneal sarcoma patients but noted a higher risk of complications. They included various histological types and had a small sample size, and did not clearly balance baseline characteristics between different treatment groups, which may have led to different results from ours (23). Falk Roeder et al. demonstrated the benefits of IORT, and although their sample size was larger (156 patients), they only compared IORT with combined treatment (IORT combined with additional radiotherapy), without analyzing the group of patients not receiving IORT, leaving the benefits of IORT unclear (24).

Previous studies often included multiple histological types such as liposarcoma, leiomyosarcoma, malignant fibrous histiocytoma, etc. and did not specifically analyze liposarcoma. Most studies had limitations such as small sample sizes and unbalanced baseline characteristics. Additionally, some studies had design flaws, making it difficult to clearly distinguish between the effects of using IORT and not using IORT. Our study used a larger cohort specifically focusing on liposarcoma and balanced baseline characteristics through 1:1 PSM, making the cohorts more comparable. Additionally, we conducted a detailed analysis of the effects of IORT by comparing patients who received IORT to those who did not, those who received only IORT to those who received no radiotherapy at all, and those who received IORT in combination with other adjuvant radiotherapies to those who received only other adjuvant radiotherapies, eliminating interference from preoperative/postoperative adjuvant radiotherapy. However, the results indicated that receiving intraoperative radiotherapy had no impact on patient prognosis, regardless of whether adjuvant radiotherapy was administered.

This investigation aimed to examine the effects of IORT on the prognosis of RPL patients and suggest novel approaches for managing the entire process for these patients. Regrettably, our findings imply that IORT does not hold significant value for the prognosis of these patients. Based on the outcomes of numerous retrospective studies and the STRASS experiment, it is not recommended to use IORT as a routine treatment for RPL patients, because the effect of combined or single use of IORT is the same as that of other radiotherapy modalities. What’s more, preoperative radiotherapy has the advantage that the target (tumor volume [GTV]) is clearly visible and can be more precisely defined to ensure repeatability and accuracy of the radiotherapy plan, and lower and, therefore, safer radiation doses are used before surgery (5). In conclusion, preoperative radiation might be the best option.

Moreover, previous studies have mainly reported LC as the primary endpoint for the benefit of IORT. While it is possible that IORT enhances LC, it may not translate into better OS due to some factors, leading our results to contradict some previous results. It is important to note, however, that our study results were influenced by a selection bias in the patients chosen for IORT. Our study is a retrospective exploratory research, and the results offer insight into future radiotherapy methods for RPL patients. Further prospective experiments could provide a better analysis of IORT’s efficacy.

The subsequent generation of STRASS 2 trials will assess the influence of neoadjuvant chemotherapy in leiomyosarcoma and high-risk liposarcoma patients, thereby promoting the integrated treatment of RPS (25).

Our study has several limitations. The SEER database only includes U.S. data, potentially introducing selection bias by excluding patients treated elsewhere. It also lacks key treatment details such as surgical margins, resection extent, and radiotherapy doses, limiting our analysis, especially given the importance of surgical margins. Additionally, SEER’s limited survival data prevents assessment of local control and recurrence rates, and the absence of TNM staging restricts patient staging analysis. As a retrospective study, inherent selection biases exist despite using 1:1 PSM to mitigate these. Nevertheless, SEER remains valuable for studying rare tumors and overcoming sample size limitations.

## Conclusion

Our results suggest that IORT alone or combined with pre - or post-operative radiotherapy does not improve patients’ OS. As an exploratory study, although the sample size is small and public data is used, we have conducted the first global exploration of the efficacy of IORT in RPL, which is expected to provide references for further prospective clinical research. Future studies should include prospective and randomized controlled trials. Additionally, further multicenter studies could not only increase the sample size but also enhance the representativeness and external validation of the research.

## Data Availability

The original contributions presented in the study are included in the article/supplementary material, further inquiries can be directed to the corresponding authors.
